# Bibliometric Studies and the Discipline of Social Media Mental Health Research. Comment on “Machine Learning for Mental Health in Social Media: Bibliometric Study”

**DOI:** 10.2196/28990

**Published:** 2021-06-17

**Authors:** Philip Resnik, Munmun De Choudhury, Katherine Musacchio Schafer, Glen Coppersmith

**Affiliations:** 1 Department of Linguistics and Institute for Advanced Computer Studies University of Maryland College Park, MD United States; 2 School of Interactive Computing Georgia Institute of Technology Atlanta, GA United States; 3 Department of Psychology Florida State University Tallahassee, FL United States; 4 Qntfy Boston, MA United States

**Keywords:** bibliometric analysis, machine learning, mental health, social media, bibliometrics

Bibliometric studies like the recent article by Kim et al [1] in the *Journal of Medical Internet Research* play an essential part in understanding the evolution of emerging, fast-moving research on machine learning for mental health in social media. However, the intended value of this paper’s contribution is tempered by some important lessons it teaches us about the current state of research on this topic.

The first key lesson is that computationally oriented research on mental health remains highly fragmented. Notably, variants on the cover term “mental health” are included in the illustrative search query but, crucially, “clinical psychology” and “psychiatry” are not. The terminological difference here reflects a prevailing technological focus often separated from clinical research and even more distant from clinical practice. Kim et al [1] do discuss a trend toward clinically validated self-report questionnaires to gather clinically relevant information. However, the review’s overall approach, from the search terms to the keyword analysis, simultaneously reflects and reinforces a widespread technological disregard for basic considerations in clinical psychology and psychiatry, such as the distinction between the symptoms of the disorders versus the disorders themselves. As technologists, we are often happy just to get our hands on enough data to work with. However, real progress toward solving these important problems demands a more careful definition of the actual mental health constructs under investigation and greater attention to the question of validity [2,3], with research questions and experimental choices guided by knowledge of the subject domain.

Second, the inclusion terms reflect a widespread narrow focus on methods, such as “neural network” and “hybrid intelligent system,” rather than the problems for which those methods are contributing solutions, such as “screening,” “risk assessment,” or “monitoring.” Even the cover term “natural language processing” focuses narrowly on engineering versus “computational linguistics” as a parent scientific discipline. Further lacking in the methodology-centric perspective are searches based on theoretical frameworks (which guide research, treatment, and intervention) or DSM-5 (Diagnostic and Statistical Manual of Mental Disorders, 5th Edition) diagnoses (eg, major depressive disorder or persistent depressive disorder versus “depression,” which is not a diagnosis). The review reflects and reinforces a general tendency to frame machine learning research in terms of technical “tasks” rather than connecting them more directly with real-world problems, a necessary step toward translating technological progress into the broader mental health ecosystem within which the technology will ultimately need to be situated [4,5].

Third, the bibliometric approach taken here reflects a traditional top-down view that fails to break down information silos in a rapidly evolving field. It is now standard to cast the net more broadly by searching for citations in resources like Google Scholar and/or looking at papers’ references (cf Franklin et al [6]), and then narrow using exclusion criteria. Such practices can illuminate the wider space of relevant search terms and sources—for example, the notable absence of suicidality here among mental conditions, at least in the illustrative search—and uncover unexpected connections. Even within the most rigorous meta-analysis frameworks (Moher et al [7]), studies can miss “gray literature” (eg, conference proceedings, preprints, collected data that have never been analyzed, presented on, or published). For example, the substantially similar prior study by Chancellor and De Choudhury [3] needed to adjust for the limitations of indexing services, which had large gaps for conferences known to be important in this research area (eg, Association for the Advancement of Artificial Intelligence [AAAI], Association for Computational Linguistics [ACL], Association for Computing Machinery [ACM], Neural Information Processing Systems [NIPS/NeurIPS], American Medical Informatics Association [AMIA])—they were careful in particular to include the Workshop on Computational Linguistics and Clinical Psychology (CLPsych), a key interdisciplinary publication venue for natural language processing, machine learning, and mental health since 2014.

Kim et al [1] are to be commended for undertaking a bibliometric study with the goal of advancing our understanding of machine learning for mental health in social media. However, we would encourage thinking about their article as a different kind of contribution, even if not the intended one: it is an opportunity to draw attention to an increasing need, as the field grows, to approach this research space not only as technologists, but also as partners with clinical researchers and clinicians.

## Abbreviations

AAAI: Association for the Advancement of Artificial Intelligence
ACL: Association for Computational Linguistics
ACM: Association for Computing Machinery
AMIA: American Medical Informatics Association
CLPsych: Computational Linguistics and Clinical Psychology
DSM-5: Diagnostic and Statistical Manual of Mental Disorders, 5th Edition
NIPS/NeurIPS: Neural Information Processing Systems
